# Cancer Associated Fibroblasts: Naughty Neighbors That Drive Ovarian Cancer Progression

**DOI:** 10.3390/cancers10110406

**Published:** 2018-10-29

**Authors:** Subramanyam Dasari, Yiming Fang, Anirban K. Mitra

**Affiliations:** 1 Medical Sciences Program, Indiana University School of Medicine, Bloomington, IN 47401, USA; sudasari@iu.edu (S.D.); yimfang@iu.edu (Y.F.); 2 Indiana University Melvin and Bren Simon Cancer Center, Indianapolis, IN 46202, USA; 3 Department of Medical and Molecular Genetics, Indiana University School of Medicine, Indianapolis, IN 46202, USA

**Keywords:** ovarian cancer, tumor microenvironment, cancer associated fibroblasts, fibroblast, cross-talk, invasion, angiogenesis, ECM, chemoresistance, therapy

## Abstract

Ovarian cancer is the most lethal gynecologic malignancy, and patient prognosis has not improved significantly over the last several decades. In order to improve therapeutic approaches and patient outcomes, there is a critical need for focused research towards better understanding of the disease. Recent findings have revealed that the tumor microenvironment plays an essential role in promoting cancer progression and metastasis. The tumor microenvironment consists of cancer cells and several different types of normal cells recruited and reprogrammed by the cancer cells to produce factors beneficial to tumor growth and spread. These normal cells present within the tumor, along with the various extracellular matrix proteins and secreted factors, constitute the tumor stroma and can compose 10–60% of the tumor volume. Cancer associated fibroblasts (CAFs) are a major constituent of the tumor microenvironment, and play a critical role in promoting many aspects of tumor function. This review will describe the various hypotheses about the origin of CAFs, their major functions in the tumor microenvironment in ovarian cancer, and will discuss the potential of targeting CAFs as a possible therapeutic approach.

## 1. Introduction

Ovarian cancer is the deadliest of all the gynecologic malignancies, and is the fifth leading cause of cancer related deaths among women in the United States. There has been only a modest improvement in ovarian cancer patient prognosis over the last several decades [1,2]. Therefore, there is a critical need for focused research to improve our understanding of the disease and develop novel therapies that are more effective. In the past, most of the research efforts were focused on the cancer cells in isolation. However, recent research has identified the tumor microenvironment as a key factor in promoting cancer progression [3,4,5,6,7]. The cancer cells recruit various normal cells and reprogram them to produce factors beneficial to tumor growth and spread. These normal cells present within the tumor constitute the tumor stroma and can compose 10–60% of the tumor volume [3].

In order to survive and proliferate, the cancer cells productively interact with their microenvironment. The tumor microenvironment is complex and contains a variety of cells constituting the tumor stroma. Tumors however, can only grow if their complex tissue environment provides them with a milieu of factors and conditions that can sustain their growth and spread [8]. A complicated bidirectional interaction is therefore happening at the interface between the genetically unstable malignant cells and the genetically stable stroma, a process that will determine the degree of tumor promotion and proliferation, invasiveness, potential for spread, and even patient prognosis [9].

The tumor stroma consists of cellular components like the cancer associated fibroblasts (CAFs), immune cells, endothelial cells, pericytes, adipocytes, and so forth, as well as acellular components like the extra cellular matrix proteins (ECMs) [3,4,5,6,7]. Each of these tumor microenvironmental elements has been shown to play important roles in tumor growth and progression in various cancers, including ovarian cancer. CAFs are an important constituent of the tumor stroma, and this review will focus on providing an overview of the origin, function, and potential targeting of CAFs in ovarian cancer therapy.

## 2. Origin of CAFs

**Figure 1:**
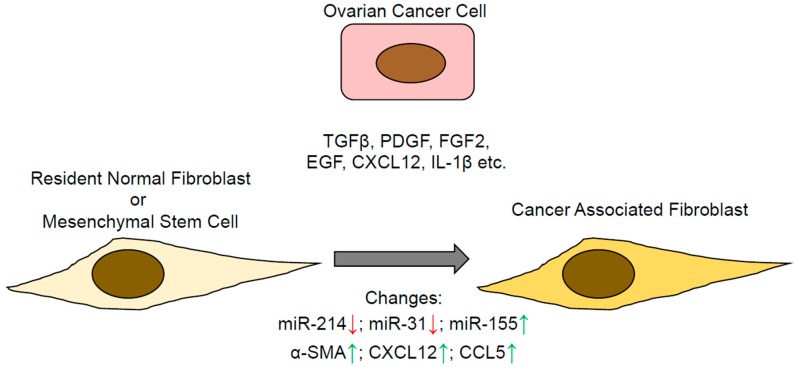
Formation of cancer associated fibroblasts (CAFs) through the reprogramming of resident normal fibroblasts or mesenchymal stem cells by ovarian cancer cells. TGF-β: transforming growth factor beta; PDGF: platelet-derived growth factor; FGF: fibroblast growth factor; EGF: epidermal growth factors; CXCL12: C-X-C Motif Chemokine Ligand 12; CCL5: C-C Motif Chemokine Ligand 5; ↑: upregulated;↓: downregulated.

There are several hypotheses about the origin of CAFs, which include the reprogramming of the resident normal fibroblasts by the cancer cells, and differentiation of mesenchymal stem cells (Figure 1). A less widely accepted theory is that they are already present as a small subpopulation of normal fibroblasts, which are selected for by the cancer cells [10]. These subpopulations may have acquired a mutation, or epigenetic alterations, independent of the tumor cells, which transform them into activated fibroblasts. A proinflammatory microenvironment resulting in the generation of reactive oxygen species may promote acquisition of genetic lesions [11]. As the tumor develops in their vicinity, these subpopulations might be selected for by the cancer cells for their ability to support tumor growth [10]. Since mutations are not commonly found in the tumor stroma, and CAFs are not believed to have clonal populations with distinct genetic changes, this hypothesis has not gained much traction [12]. Fibroblasts are mesenchymal cells that are generally present in the basement membrane and serve as a scaffold, secreting ECMs, and growth and trophic factors [13,14]. They are generally in a quiescent “inactive” state, but retain some plasticity, and are capable of getting “activated” by various physiological stimuli. During development, dermal fibroblasts play a role in tissue patterning, and can differentiate into multiple kind of cells, including hair follicle cells, papillary cells, reticular cells, and pre-adipocytes [15]. The fibroblasts get activated at the site of wound healing by factors such as insulin-like growth factors (IGFs), transforming growth factor beta 1,2,3 (TGF-β 1,2,3), and platelet-derived growth factor (PDGF), among others. These activated fibroblasts express α-smooth muscle actin (α-SMA), which makes them contractile, and helps in wound closure. These α-SMA expressing fibroblasts are called myofibroblasts. They secrete various ECMs and extracellular proteases, which help in the initial wound healing and development of the scar. They also secrete factors like TGF-β to stimulate epithelial to mesenchymal transition in the epithelial cells around the wound. This enables the epithelial cells to move and close the wound. Thereafter, as the wound heals, epithelialization is promoted by epidermal growth factors (EGFs) and the keratinocyte growth factor (KGF) produced by the fibroblasts [16].

CAFs display several traits of the activated fibroblasts found in healing wounds, including upregulation of TGF-β, increased secretion of ECMs, extra cellular proteases, and expression of α-SMA. It is believed that cancer cells can recruit the resident normal fibroblasts and reprogram them into CAFs. Several reports have demonstrated evidence in support of this hypothesis in ovarian cancer [7]. Ovarian cancer cells produce factors including TGF-β and PDGF, that can change normal fibroblasts into “activated” CAFs. We have previously shown that ovarian cancer cells can induce a change in expression of a set of 3 microRNAs in the resident normal omental fibroblasts, which reprograms them into CAFs [7]. miR-214 and miR-31 were found to decrease, while miR-155 expression increased, in the normal fibroblasts because of their interaction with the metastasizing ovarian cancer cells. This resulted in their reprogramming into CAFs (Figure 1). It was the first report of a set of microRNAs reprogramming normal fibroblasts into CAFs. Simultaneous inhibition of miR-214 and miR-31, along with overexpression of miR-155, could convert normal fibroblasts into CAFs. These results supported previous findings in other cancers, which demonstrated the absence of mutations in CAFs. Moreover, CAFs can be isolated from tumors and cultured in vitro for several passages, and yet retain their phenotype and their ability to support cancer cell functions. This suggested a potential role of epigenetic regulation [17]. The role of microRNAs in reprogramming of normal fibroblasts into CAFs further revealed a potential mechanism. Interestingly, overexpressing miR-214 and miR-31 and inhibiting miR-155 simultaneously in CAFs could revert them back into normal fibroblasts. This offers a potential opportunity to normalize a key component of the tumor microenvironment. Research on targeting the tumor microenvironment has revealed that the normalization of the tumor microenvironment is a more effective approach as compared to attempts at obliterating it altogether. The latter typically leads to the cancer cells becoming more aggressive. Depleting α-SMA positive CAFs in a transgenic mouse model of pancreatic ductal adenocarcinoma through induction of thymidine kinase by ganciclovir administration, either early in the tumor precursor stage or late carcinoma stage, led to the development of undifferentiated tumors, which were highly invasive and resulted in decreased survival [18]. Since this was observed when the CAFs were ablated in the precursor lesions or in the late carcinomas, it indicated that irrespective of tumor stage, in the absence of the microenvironmental support, the more aggressive cancer cell clones are selected. These findings are similar to the increased metastasis observed upon pericyte depletion [19].

Other hypotheses about the origin of CAFs include the recruitment of mesenchymal stem cells by the cancer cells to the tumor [10,20]. Human pancreatic cancer cells were shown to recruit bone marrow derived progenitors when injected in mice [21]. The resulting tumors had about 40% myofibroblasts derived from bone marrow cells. Similarly, CAFs were shown to be derived from mesenchymal stem cells in ovarian cancer and supported tumor growth through the secretion of the paracrine factor IL-6 [22]. Ovarian cancer cells have been reported to secrete IL-1β that leads to the decreased expression of p53 protein in the ovarian fibroblasts, converting them into CAFs [23]. The decreased p53 resulted in increased secretion of IL-8, growth regulated oncogene-alpha (GRO-α), IL-6, IL-1β, and vascular endothelial growth factor (VEGF) by the CAFs. Mesenchymal stem cell derived CAFs were also shown to regulate ovarian cancer stem cells through bone morphogenetic protein secretion, which resulted in resistance to chemotherapy [24,25]. The mesothelial cells lining the peritoneum and the omentum have also been reported as a source of CAFs in ovarian cancer peritoneal and omental metastasis [26]. The mesothelial cells have been shown to undergo mesothelial to mesenchymal transition under the influence of ovarian cancer cell secreted TGF-β, which can form a subpopulation of the CAFs in ovarian cancer metastatic tumors [27]. Others have demonstrated that ovarian cancer cells interact with the mesothelial cells in a β1-integrin-dependent manner to induce mesothelial to mesenchymal transition and convert them into CAFs [28].

## 3. CAF Markers

Since CAFs in the tumor microenvironment are functionally very similar to the activated fibroblasts in healing wounds, they both share several markers. CAFs express α-SMA, which is also a marker of myofibroblasts in wound healing [10]. Ovarian cancer CAFs also express α-SMA, while normal fibroblasts do not [29]. However, the expression of α-SMA is only one of many changes that occur in activated fibroblasts [30]. The levels of expression of α-SMA may also vary between CAFs. As evidenced by the findings of Mhawech-Fauceglia et al., the CAFs in ovarian tumors are predominantly α-SMA positive, but not all of them stain for the protein [29]. This indicates the existence of a certain level heterogeneity within the CAF population.

In addition to α-SMA, many other markers have been reported to distinguish CAFs from normal fibroblasts. They include fibroblast activated protein (FAP), S100A4, and platelet derived growth factor receptor, among others [7,29,31]. FAP, a cell surface serine protease, has emerged as a specific marker of CAFs in ovarian cancer [32]. While each of them has been shown to be an effective marker by different groups, there is no clear consensus about a universal marker for CAFs. The probable reason for this is that CAFs are a heterogeneous population, with some expressing one marker and others expressing other markers. α-SMA and PDGF positive CAFs do not overlap with S100A4 positive CAFs in pancreatic cancer [33]. The mutual exclusivity and heterogeneity in CAF marker expression may impart unique functions; for example, FAP and podoplanin positive CAFs were found to be immunosuppressive through a nitric oxide-dependent mechanism, while FAP positive and podoplanin negative CAFs were not immunosuppressive in lung adenocarcinoma [34]. For prostate cancer, CAFs expressing high CD90 had greater tumor promoting capacity than CAFs expressing low CD90 [35]. Pancreatic ductal adenocarcinomas have a subpopulation of CAFs that are distinct from those expressing α-SMA. These CAFs express proinflammatory mediators like IL-6, and mediate a paracrine interaction with the carcinoma cells [36]. In ovarian cancer, the expression levels of different CAF markers, CD10, podoplanin, FAP, Platelet-derived growth factor receptor alpha (PDGFRα), Platelet-derived growth factor receptor beta (PDGFRβ), S100 calcium binding protein A4 (S100A4), α-SMA, snail family transcriptional repressor 2 (SNAI2, commonly known as Slug), Zinc finger E-box-binding homeobox 1 (ZEB1), and twist family bHLH transcription factor 1 (TWIST1), clustered the CAFs into different subgroups showing different protein expression patterns [31]. 

Due to the continuous reciprocal interactions of CAFs with cancer cells, it is quite possible that the CAFs can undergo dynamic changes in their marker expression and functions depending on the heterogeneity of the cancer cells within the tumors. A recent study identified a unique subset of CAFs expressing the metallo-endopeptidase CD10 and the complement anaphylatoxin receptor GPR77 [37]. These CAFs were enriched following neoadjuvant chemotherapy, and were shown to promote cancer stem cell self-renewal through the secretion of IL-6 and IL-8. Therefore, their abundance in the tumors of breast cancer patients predicted a poor prognosis. Similarly, the evolving ovarian cancer metastatic tumors are metabolically reprogrammed by CAFs through the secretion of C-C Motif Chemokine Ligand 5 (CCL5), C-X-C motif chemokine ligand 10 (CXCL10), and IL-6 to utilize glycogen [38]. The CAFs with activated p38 MAP kinase signaling were capable of inducing such reprogramming. Therefore, considering the heterogeneity of CAFs, it is important to take into account their functional effects in promoting tumor progression as well as the potential of dynamic changes in them.

## 4. CAF Functions

**Figure 2:**
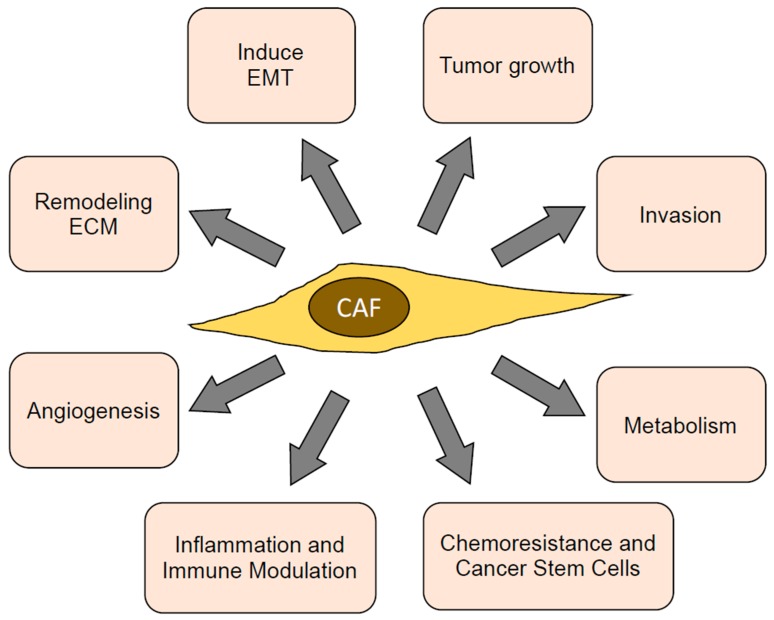
Functions of CAFs contributing towards tumor progression. ECM: extra cellular matrix; EMT: epithelial–mesenchymal transition.

CAFs have multiple functions in the tumor microenvironment, which directly or indirectly promote tumor progression. Most of these functions are mediated through the secretion of paracrine factors, ECMs, and proteases, as well as through cell surface receptors and direct contact with cancer cells. These functions and their underlying mechanisms are detailed below, outlined in Figure 2, and listed in Table 1.

### 4.1. Promoting Tumor Growth

CAFs have predominantly been demonstrated to have tumor-promoting functions. They stimulate cancer cell survival, growth, and invasion, enhance the stiffness of the extracellular matrix, contribute to angiogenesis by releasing pro-angiogenic factors, contribute to a pro-inflammatory milieu, and impact on the activation state of various immune cells [50]. CAFs are crucial in tumor-stroma communication through modulation of the extracellular matrix, fibrogenesis, and chemoattraction of other stromal cells. Several studies have demonstrated that tumor–CAF crosstalk promotes growth and invasion of the particular cancer cells [39]. CAFs produce autocrine and paracrine cytokines that promote the growth and biological characteristics of tumors [7]. In addition to classical growth factors, including EGF and hepatocyte growth factor (HGF), novel CAF secreted proteins (secreted frizzled related protein 1, and IGF like family member (IGF) 1 and 2) and membrane molecules (integrin α11 and syndecan 1) have also been identified to possess cancer cell supporting roles. These factors directly or indirectly stimulate tumor growth and survival, or enhance their migratory and invasive properties [51]. Several secreted molecular regulators of CAFs have a pro-tumorigenic role, such as the TGF-β superfamily and bone morphogenic proteins (BMPs), PDGFs, EGFs, fibroblast growth factors (FGFs), and sonic hedgehog (SHH) [52].

Initial experiments with co-injection of CAFs with simian virus 40 (SV40)-transformed prostate epithelial cells in mice resulted in tumors resembling prostatic intraepithelial neoplasia, whereas normal fibroblasts did not. Similarly, co-transplantation of myofibroblasts with Ras-transformed hepatocytes strongly enhances tumor growth through the TGF-β/PDGF axis [39]. In addition, CAFs induce forkhead box Q1 (FOXQ1) expression; as a result, N-myc downstream-regulated gene 1 (NDRG1) is trans-activated to enhance hepatocellular carcinoma (HCC) initiation. Interestingly, pSTAT6/CCL26 signaling is induced by the FOXQ1/NDRG1 axis, thus recruiting hepatic stellate cells (HSCs), the main cellular source of CAFs, to the tumor microenvironment. Thereby, tumor-initiating properties are enhanced at least partly through a positive feedback loop between CAFs and HCC cells [53]. Taken together, these indicate that CAFs can provide growth-promoting signals to epithelial cells, and support epithelial transformation [54].

Tumor-derived TGF-β1 has been reported to activate tumor stroma, and thereby facilitate tumor growth and progression. Inhibition of the TGF-β pathway in mouse fibroblasts through conditional inactivation of the *TGFBR2* gene is associated with increased oncogenic potential of the adjacent epithelia [55]. An invasive breast cancer cohort study, using a randomized tamoxifen trial, demonstrated that TGF-β receptor type-2 expression in cancer-associated fibroblasts regulates breast cancer cell growth and survival, and is a prognostic marker in pre-menopausal breast cancer [56]. Mesenchymal stem cell derived CAFs recruited to the stroma of the dysplastic stomach express IL-6, Wnt5a, and bone morphogenetic protein 4, which promote tumor growth through DNA hypomethylation [57]. In oral squamous cell carcinoma (OCC), CAFs promote the production of endogenous reactive oxygen species (ROS) through CCL2 expression, which induces the cell cycle regulatory proteins, and promotes OCC proliferation, migration, and invasion [58]. CAFs have also been reported to promote Th2 polarization of the tumor microenvironment, and stimulate tumor growth and metastasis by recruiting tumor-associated macrophages (TAMs), myeloid derived suppressor cells (MDSCs), and T regulatory cells (T_regs_) [8,59].

In ovarian cancer, CAFs promote tumor invasion and growth through the secretion of a number of chemokines, cytokines, and growth factors like CCL5, IL-6, IL-8, HB-EGF, and TGF-α, among others [7]. These secreted factors were regulated by the decreased expression of miR-214 and miR-31, and an increased expression of miR-155, in CAFs induced by ovarian cancer cells. CCL5 was a target of miR-214 and miR-31, and was responsible for homing of the ovarian cancer cells onto plugs of CAFs in vitro [7]. Inhibiting CCL5 with a neutralizing antibody was sufficient to reduce tumor growth of co-injected CAFs and ovarian cancer cells in mice [7].

### 4.2. Promoting Tumor Invasion

Tumor invasion is a key hallmark of cancer and is essential for successful dissemination of the cancer cells. Myofibroblasts have the inherent ability to invade through the ECM in the basement membrane during wound healing. Similarly, CAFs have the ability to invade through matrix, and have been widely reported to promote invasiveness of cancer cells [3]. There are several potential mechanisms by which CAFs can directly or indirectly promote cancer cell invasiveness. These include secretion of factors and proteases that help in the invasion. Zhu et al. (2016) [40] reported that Gal-1-regulated CAF activation promotes breast cancer cell metastasis by upregulating MMP-9 expression in breast cancer. Recent studies have shown that breast CAFs overexpress the chemokine CXCL1, a key regulator of tumor invasion and chemo-resistance. TGF-β negatively regulates CXCL1 expression in CAFs through Smad2/3 binding to the promoter, and through suppression of HGF/c-Met autocrine signaling [60]. CAFs can also induce changes in the cancer cells, which helps in their invasiveness. They have been reported to promote the metastatic activity of breast cancer cells by activating the transcription of HOTAIR via TGF-β1 secretion [61].

CAFs can serve as engines for collective invasion of directly interacting cancer cells through heterotypic interactions between the N-cadherin expressed on CAFs with the E-cadherin on cancer cells [62]. Interestingly, a dual mechanism is involved. CAFs favor invasion of cancer cells by pulling them away from the tumor, while cancer cells further enhance their spread by polarizing CAF migration away from the tumor. Along similar lines, vimentin is reported to be necessary for lung adenocarcinoma metastasis by maintaining heterotypic tumor cell–CAF interactions during collective invasion [63]. Cdc42EP3—a member of the BORG family of Cdc42 effectors—is highly expressed in CAFs, and regulates the actin and septin fibrillar networks. Coordination between the actin and the septin networks in CAFs is required for force-mediated matrix remodeling, promoting cancer cell invasion, angiogenesis, and tumor growth [64].

In ovarian cancer we have previously shown that CAFs can promote coordinated invasion of the cancer cells, which promotes metastasis [7]. Using a novel 3D live confocal imaging-based co-invasion assay, we observed that the CAFs derived from ovarian cancer omental metastasis are able to closely interact with ovarian cancer cells and invade through matrigel by forming distinct networks of CAFs and cancer cells, which invaded together. Cancer cells alone invaded at a slower rate and failed to form the network consisting of cellular associations. The mechanism of CAF–cancer cell interactions reported by Labernadie et al. [62] involving heterophilic E-cadherin/N-cadherin junctions could potentially be playing a role in these interactions between the CAFs and ovarian cancer cells. A comparison of gene expression profiles of CAFs from omental metastasis with normal omental fibroblasts revealed that the CAFs secrete multiple chemokines and cytokines that can potentially regulate invasion and motility of cancer cells [7]. Among them, CCL5 was identified as a key CAF derived mediator of metastasis, which was itself regulated by miR-214 and miR-31. Both these microRNAs are downregulated in CAFs during the reprogramming of normal fibroblasts by metastasizing ovarian cancer cells [7]. Zhao et al. (2017) identified STAT4 as a key regulator of ovarian cancer metastasis via Wnt7a-induced activation of CAFs [65]. The concomitant increased production of CXCL12, IL6, and VEGFA by CAFs within the tumor microenvironment could enable peritoneal metastasis of ovarian cancer via induction of the EMT program. They also established a model of promotion of ovarian cancer metastasis by STAT4 via tumor-derived Wnt7a-induced activation of CAFs [65]. CAFs promote ovarian cancer cell proliferation, migration, and invasion through the paracrine FGF-1 factor. The FGF-1/FGFR-4 signaling axis regulates the stromal microenvironment in ovarian carcinomas. CAFs also activate the expression of Snail1 and MMP3, as well as reduce the expression of E-cadherin [66].

### 4.3. Inducing EMT in Cancer Cells 

An epithelial-mesenchymal transition (EMT) is a biological process that allows a polarized epithelial cell, which normally interacts with the basement membrane via its basal surface, to undergo multiple biochemical changes that enable it to assume a mesenchymal cell phenotype, which includes enhanced migratory capacity, invasiveness, elevated resistance to apoptosis, and greatly increased production of ECM components. The completion of an EMT is signaled by the degradation of the underlying basement membrane and the formation of a mesenchymal cell that can migrate away from the epithelial layer in which it originated [67]. The idea that epithelial cells can downregulate epithelial characteristics and acquire mesenchymal characteristics arose in the early 1980s from observations made by Elizabeth Hay [68]. Over the subsequent years, the importance of EMT in cancer progression has been well established. The heterotypic interactions of cancer cells with the microenvironment, including CAFs, has been shown to be a key inducer of EMT.

Coculturing CAFs with lung cancer cells can induce miR-33b downregulation and promote epithelial cells EMT. miR-33b overexpression in lung cancer cells can counteract CAF-induced EMT. Interestingly, Snail1 expression in fibroblasts activates the inductive effects of CAFs on lung cancer cells. Snail1-expressing cancer-associated fibroblasts induce lung cancer cell epithelial-mesenchymal transition through miR-33b [41]. CAF conditioned medium induced epithelial-mesenchymal transition (EMT) by regulating the expression of EMT-associated markers E-cadherin and vimentin, and modulated metastasis-related genes MMP-2 and VEGF, both in vitro and in vivo. Further studies demonstrated that CAFs enhanced the metastatic potential of lung cancer cells by secreting IL-6 and subsequently activating the JAK2/STAT3 signaling pathway [42]. TGF-β1 secreted by CAFs can induce EMT in the interacting cancer cells and promote metastasis [69,70]. In ovarian tumors, CAF derived exosomes contain higher levels of TGF-β1 compared to those derived from normal omental fibroblasts [71]. Theses exosomes, upon uptake by ovarian cancer cells, induce EMT through the activation of SMADs. Activation of STAT3 by microenvironmental IL-6 can also induce EMT in ovarian cancer cells [72]. CAFs were reported to be the major source of IL-6 in the tumor microenvironment of ovarian tumors [72]. Therefore, CAFs can make cancer cells more aggressive by inducing EMT in them through various paracrine mechanisms.

### 4.4. Remodeling the ECM

Every organ has an ECM with unique composition, providing physical support for tissue integrity and elasticity. It is a dynamic structure that is constantly remodeled to control tissue homeostasis [73]. Dysregulation of ECM composition, structure, stiffness, and abundance contributes to several pathological conditions, such as fibrosis and invasive cancer. Typically, tumors have much stiffer ECMs, causing altered dynamics of the biophysical and biochemical interactions of the cancer cells with their microenvironment [74]. The increased stiffness of the matrix promotes invasiveness and motility of the cancer cells through improved invadosome and lamella formation [75]. Matrix stiffness drives EMT and metastasis through the TWIST1–G3BP2 mechanotransduction pathway in breast cancer [76]. A 9-gene matrisome gene signature has been identified through the analysis of available databases, and is associated with tumor progression through promotion of EMT, angiogenesis, hypoxia, inflammation, and altered metabolism in several cancers, including ovarian cancer [43]. Altered ECMs and ECM remodeling enzymes, like matrix metalloproteinases (MMPs), tissue inhibitors of metalloproteinases (TIMPs), and lysyl oxidases (LOXs), work to create a stiffer microenvironment permissive for tumor cell growth, migration, and invasion [44]. The increased secretion of fibronectin and LOXs by breast cancer CAFs contribute towards the remodeling of the ECMs in these tumors, promoting invasion and metastasis [77]. TGF-β activates the secretion of versican by CAFs in high-grade serous ovarian tumors, which induces the expression of MMP9 and CD44 by the cancer cells, resulting in ECM remodeling and invasion [78]. A recent study has demonstrated the role of ovarian cancer cell derived inhibin βA in effectively inducing CAFs, which then secrete increased amounts of collagens and other ECMs [79]. Therefore, CAFs serve the important function of remodeling the ECMs through altered secretion of the matrisome components, and provide the ideal microenvironmental stiffness for tumor progression.

### 4.5. Inducing Angiogenesis 

As the tumor grows, the cancer cells are further removed from the existing blood vessels, and as a result, experience depleted levels of oxygen and nutrients. This typically places a limit to the tumor size, as cell proliferation in the regions well supplied by the blood vessels is balanced by cell death in the regions deprived of oxygen and nutrients. Therefore, in order to progress, the tumors must induce angiogenesis. It is the formation of a new vascular network to supply nutrients and oxygen, and remove waste products. Multiple factors, like VEGF, basic fibroblast growth factor (bFGF), interleukin-8 (IL-8), placenta-like growth factor (PLGF), TGF-β, platelet-derived endothelial growth factor (PD-EGF), pleiotrophin, activated hypoxia-inducible factor-1α (HIF-1α), and so forth, have been shown to trigger angiogenesis [80,81]. Many of these pro-angiogenic factors are contributed by the tumor microenvironment [82]. CAFs induce angiogenesis directly, as well as indirectly, through VEGFA, PDGFC, FGF2, CXCL12, osteopontin, and CSF3 secretion, ECM production, and recruitment of myeloid cells [82]. SDF-1 secreted by breast cancer CAFs has been involved in mobilization of endothelial precursor cells from bone marrow, thereby inducing de novo angiogenesis, as well as in tumor growth through a paracrine effect on CXCR4 expressing cancer cells [45]. Similarly, fibroblast-derived SDF-1 synergized with IL-8 in the promotion of a complete angiogenic response in recruited endothelial cells in pancreatic cancer [46]. SDF-1 is induced in breast cancer CAFs by oxidative stress-mediated activation of HIF-1 [83]. Chloride intracellular channel protein 3 (CLIC3) secreted by breast cancer CAFs induces angiogenesis through an active transglutaminase-2 (TGM2) mediated mechanism [84]. MMP-13 secreted by CAFs promotes tumor angiogenesis by releasing VEGF entrapped in the ECM, thereby leading to increased invasion of endothelial cells in squamous cell carcinoma and melanoma [85]. Ovarian cancer CAFs can indirectly induce angiogenesis through increased secretion of pro-inflammatory factors IL-6, COX-2, and CXCL1 [86]. Ovarian cancer cell expression HOXA9 induces CAFs to secrete CXCL12, IL-6, and VEGF-A expression, which promotes angiogenesis [87]. Ovarian cancer CAFs have also been shown to induce angiogenesis by secreting VEGF-C as a result of induction by Sonic Hedgehog (SHH) from ovarian cancer cells [88]. While angiogenesis can be induced by cancer cells as well as the tumor microenvironment, CAFs are important direct or indirect contributors to the process, and hence towards cancer progression.

### 4.6. Inflammation and Immune Modulation 

Inflammation is a normal physiological response that is initiated in injured tissue and helps in its healing. In clinical settings, chronic inflammation and cancer are closely related, and cancer is referred to as “wounds that never heal”. During tissue injury associated with wounding, cell proliferation is enhanced while the tissue regenerates; proliferation and inflammation subside after the assaulting agent is removed or the repair completed. In contrast, proliferating neoplastic cells continue to proliferate in microenvironments rich in inflammatory cells, and growth and survival factors, that support their growth [89]. Pro-inflammatory cytokines are secreted by cancer cells and CAFs to attract immune cells to the tumor. Macrophages actively attracted into tumor regions along defined chemotactic gradients start to differentiate into tumor-associated macrophages (TAMs), which further enhance the growth and metastasis of cancer cells [52]. CAFs are functionally required for mediating inflammation during squamous cell carcinogenesis, starting at the earliest pre-neoplastic stages [90]. A recent paper demonstrated that CAFs associated to incipient neoplasia exhibit a pro-inflammatory signature, leading them to mainly overexpress SDF-1, IL-6, and IL-1β, as well as to recruit proangiogenic macrophages. This gene set is under the transcriptional control of nuclear factor-κB (NF-κB) and cyclooxygenase 2 (COX-2), thereby strengthening the link between CAFs and inflammatory mediators in tumor progression [47]. We have demonstrated that ovarian cancer CAFs produce an array of chemokines and cytokines, which can potentially induce a proinflammatory response [7]. These chemokines and cytokines were directly or indirectly regulated by miR-214, miR-31, and miR-155 in CAFs [7]. Several other groups have also shown many chemokines and cytokines, including IL-6, COX-2, and CXCL1, to be involved in tumor-related inflammation in epithelial ovarian cancer [50,91].

CAFs in the tumor microenvironment exert immunomodulatory effects through secretion of immunomodulatory factors that polarize responsive immune populations, such as macrophages [92]. In order for the tumor to survive, any immune response directed toward the tumor cells needs to be suppressed [52]. CAFs play important roles in shaping the tumor immunosuppressive microenvironment in oral squamous cell carcinoma by inducing the protumor M2 macrophages [93]. Immunosuppressive activity of CAFs has been reported in head and neck squamous cell carcinoma through increased expression levels of IL-6, CXCL8, and TGF1 [94]. Genetic ablation of Chitinase 3-like 1 (Chi3L1) in fibroblasts in vivo attenuated tumor growth, macrophage recruitment, and reprogramming to an M2-like phenotype, enhanced tumor infiltration by CD8+ and CD4+ T cells, and promoted a Th1 phenotype. These results indicate that CAF-derived Chi3L1 promotes tumor growth and shifts the balance of the immune milieu towards type 2 immunity [95]. Activation of the PD1/PDL1 signaling pathway in T-cells leads to T-cell exhaustion and immune suppression. IL6 secreted by CAFs in hepatocellular carcinoma activates neutrophils in the tumor microenvironment, and induces PDL1 expression in them through the JAK-STAT3 pathway. The PDL1 expressing neutrophils inhibit T-cell mediated immunity and create a protumor microenvironment [96]. Being the most abundant cellular component of the stroma, CAFs can exert their effects indirectly on tumor progression through secreted factors that help evolve a proinflammatory and immunosuppressive microenvironment for the cancer cells to thrive in.

### 4.7. Promoting Chemoresistance and Cancer Stem Cells

The eventual development of chemoresistance is the cause of most ovarian cancer related mortalities. The role of the tumor microenvironment in this process has generated great interest in recent years. Glutathione and cysteine released by fibroblasts in ovarian tumors contribute towards the depletion of platinum in the nuclei of the adjacent ovarian cancer cells, and thus impart resistance to platinum based chemotherapies [97]. CAFs can also induce therapy resistance by reducing the bioavailability of the drugs, by causing tumor microvessel leakiness. CAFs induce the *lipoma-preferred partner* (*LPP*) gene in microvascular endothelial cells through a calcium-dependent FAK/ERK/MLC2/CREB signaling pathway [48]. In one study, the increased *LPP* enhanced the endothelial cell motility and permeability through increased focal adhesions and stress fiber formation [48]. CAFs can also induce chemoresistance in cancer cells by inducing apoptosis resistance. CAFs activate STAT3 signaling in ovarian cancer cells resulting in the development of chemoresistance through the increased expression of the antiapoptotic survivin and Bcl-2 [49]. CAFs in pancreatic ductal adenocarcinoma can similarly decrease apoptosis and increase the chemoresistance of the cancer cells by the induction of transcriptional downregulation of caspases by promoter hypermethylation [98]. Ovarian cancer apoptosis was also inhibited by CAF derived exosomes that transfer miR-21 to the cancer cells. The miR-21 then downregulated its direct target apoptotic protease activating factor 1 (APAF1), conferring chemoresistance [99].

Cancer stem cells are known to be resistant to cytotoxic chemotherapy and can give rise to tumor relapse. CD10 and GPR77 expressing CAFs induce cancer stem cells in breast and lung cancer through the consistent secretion of IL-6 and IL-8 [37]. These CAFs also increase the take rate of patient derived xenograft tumors, and inhibition of GPR77 abolishes this effect [37]. In one study, autophagic CAFs in luminal breast cancer induced stemness in the cancer cells through the secretion of high-mobility group box 1 (HMGB1), resulting in the activation of toll-like receptor 4 in the cancer cells [100]. In endocrine resistant metastatic breast cancer, the transfer of CAF derived exosomes containing miR-221 activated an ER^lo^/Notch^hi^ feed-forward loop that generated CD133^hi^ cancer stem cells [101]. In ovarian cancer, the evidence of induction of cancer stem cells is limited, with a few reports indicating the role of CAF derived fibroblast growth factor 4 and IL-6 in inducing cancer stem cells [102,103]. Targeting the FHF4/FGFR2 axis that mediates the CAF-cancer stem cell signaling prevented the CAFs from inducing cancer stem cells [102]. Insulin growth factor receptor activation in ovarian cancer cells by CAFs, and the resulting increased Nanog expression, is also reported to promote cancer stem cells in ovarian cancer [104]. Overall, the potential role of CAFs in providing a stem cell niche for cancer stem cells is an idea that needs to be systematically researched.

### 4.8. Reprogramming Cancer Cell Metabolism

Cancer cells have an altered metabolism to cope with their different growth rate, nutrient availability, and the hypoxia they experience as compared to normal cells. This altered metabolism is considered one of the hallmarks of cancer, and the tumor microenvironment is a major contributor towards this phenomenon [3,4,38,105]. CAFs have been reported to secrete vesicles, which created hypoxia mimicking conditions in the cancer cells, causing reductive carboxylation of glutamine in them, and decreased oxidative phosphorylation [106]. The CAF derived vesicles are also carriers of metabolites feeding into the tricarboxylic acid cycle in the cancer cells. This brings forth a novel mechanism by which CAFs can influence the cancer cells through the transfer of metabolites and pushing away from an oxygen based energy metabolism. Using stable isotope labeling of amino acids in cell culture (SILAC) in cocultures of CAFs with ovarian cancer cells, a recent study demonstrated how the CAFs can help ovarian cancer cells switch from utilizing lipids to using glycogen reserves for energy [38]. As the metastatic tumor grows and depletes the adipocytes in the omentum, the IL-6, CXCL10, and CCL5 secreted by the CAFs induce the ovarian cancer cells to start utilizing glycogen. The activation of p38 MAP kinase in the CAFs drives the cytokine secretion, which in turn activates glycogen phosphorylase in the ovarian cancer cells [38]. This demonstrates that the dynamic changes happening in the tumor microenvironment as the tumor progresses, forces the cancer cells to reset their energy sources. CAFs can orchestrate this switch by turning off glycogen synthesis and activating glycogen utilization for glycolysis. Therefore, targeting the key enzyme in this process, glycogen phosphorylase, would be a potential therapeutic option to treat ovarian cancer metastasis.

## 5. Targeting CAFs Clinically

Since CAFs contribute towards so many critical aspects essential for tumor progression, strategies targeting CAFs to treat ovarian cancer can be potentially effective. Moreover, since CAFs themselves are genetically stable and do not have the propensity to mutate, acquiring resistance against these therapies would be less likely. Since CAFs overexpress FAP, a humanized antibody (sibrotuzumab) directed against human FAP has been tested in phase 1 clinical trials to demonstrate that it is safe to administer to patients with high levels of FAP expression in their tumors [107]. However, it did not have any beneficial effect in a phase II trial for metastatic colorectal cancer [108]. A fusion protein consisting of an anti-FAP antibody fused with IL-2 (RO6874281) is presently under clinical trials as a combination therapy with atezolizumab—an anti-PDL-1 antibody—for advanced or metastatic solid tumors (ClinicalTrials.gov: NCT03386721). In addition, a phase I clinical trial is ongoing to test RO6874281 as a single agent, or in combination with trastuzumab or cetuximab, for solid tumor, and breast, head, and neck tumors (ClinicalTrials.gov: NCT02627274). As TGF-β plays an essential role in stromal-epithelial interaction and CAF induction, targeting TGF-β is a potentially promising approach to target CAFs as well as cancer cells. Transgenic mice expressing a TGF-β antagonist were resistant to metastasis to multiple organs while not exhibiting the adverse pathological outcomes observed in TGF-β-null mice [109]. Transcription profiling of CAFs microdissected from ovarian cancer patient tumors identified a subpopulation that had activation of SMAD signaling [110]. These CAFs were markers of poor patient progression, and targeting SMAD signaling with calcitriol inhibited tumor progression in mice. At present there are as many as 60 active clinical trials on TGF-β in cancers (clinicaltrials.gov). However, it is very difficult to differentiate the role of CAF derived TGF-β from other stromal sources and cancer cell autocrine TGF-β signaling. The HGF-cMet pathway, involving the cross-talk between CAFs and cancer cells, plays a role in cancer metastasis, and is another potential target for blocking CAF–cancer cell interaction. Targeting c-Met or HGF has shown promising tumor growth inhibition and gemcitabine sensitization in vivo [111,112]. There are 69 active studies on cMet listed in clinicaltrials.gov. Targeting CAF can decrease the immunosuppressive microenvironment of the tumor, as well as lead to CD8+ T-cell activation, and enhance anti-tumor immunity [18,113].

While several strategies to target CAFs in tumors have been attempted, much remains to be studied before it can be effectively translated to the clinic. Strategies like targeting TGF-β may benefit from attacking both the cancer and stromal compartments. Importantly, the potential of combining such therapies with existing platinum and taxane based chemotherapies should be tested for ovarian cancer. However, previous experiences with targeting the tumor microenvironment have taught us that an approach towards normalization is preferable to an eradication of the tumor stroma. This is because the latter approach tends to give rise to more aggressive cancer cells. Therefore, targeting CAFs should aim for reverting them back to normal fibroblasts, rather than depleting them altogether.

## 6. Conclusions

CAFs are an important constituent of the ovarian cancer tumor microenvironment, and have been demonstrated to play an important role in tumor progression, metastasis, and chemoresistance. While a universal CAF marker has not been identified, several markers have been demonstrated in unique subpopulations, indicating that CAFs are heterogenous in this context, and this may also dictate their function. Continuous reciprocal interactions of CAFs with cancer cells and other components of the microenvironment shape their fate, marker expression, and function in the tumor. Continuing research towards a better understanding of their plasticity, regulation, function, and heterogeneity would greatly enhance the way we perceive tumors, and will determine how we treat them. Strategies to “normalize” CAFs and deprive the cancer cells of the gamut of factors provided by them may be an effective approach to complement existing therapies targeting the cancer cells.

## Figures and Tables

**Table 1:** Functional roles of cancer associated fibroblasts (CAFs) in tumor progression.

No.	Functional Role of CAF	References
1	Promoting of tumor growth	[39]
2	Promoting tumor invasion	[3,40]
3	Inducing EMT in cancer cells	[41,42]
4	Remodeling the ECM	[43,44]
5	Inducing angiogenesis	[45,46]
6	Inflammation and immune modulation	[7,47]
7	Promoting chemoresistance and cancer stem cells	[48,49]
8	Reprogramming cancer metabolism	[3,4,38]

ECM: extra cellular matrix; EMT: epithelial–mesenchymal transition.
